# Levetiracetam poisoning with acute kidney injury treated with hemodialysis: A case report

**DOI:** 10.1097/MD.0000000000030884

**Published:** 2022-09-30

**Authors:** Hiraku Sedogawa, Norihiro Watanabe, Yoshikazu Hirose, Naoko Mizouchi, Shunri Shimagaki, Hiroki Yamaguchi, Yasuo Hirose

**Affiliations:** a Department of Emergency and Critical Care Medicine, Niigata City General Hospital, Niigata, Japan; b Department of Internal Medicine, Tochigi Medical Center, Utsunomiya, Japan; c Department of Nephrology and Rheumatology, Niigata City General Hospital, Niigata, Japan.

**Keywords:** acute kidney injury, hemodialysis, levetiracetam, overdose

## Abstract

Levetiracetam is a newer second-generation anticonvulsant for the treatment of generalized and partial seizure disorders. Common adverse effects are rhabdomyolysis and neuropsychiatric problems, such as somnolence, dizziness, and mood changes. The present report describes the first case, to our knowledge, of levetiracetam overdose resulting in acute kidney injury for which hemodialysis was required.

**Patient concerns:**

A 51-year-old man presented with hypotension and disturbance of consciousness with subsequent development of oliguria and elevated creatinine. Based on his history of ingesting a large dose of levetiracetam and the course of the disease, he was considered to have been poisoned by levetiracetam.

**Diagnosis:**

Acute kidney injury induced by levetiracetam poisoning.

**Interventions/Outcomes:**

Dialysis was performed for the rapidly progressing renal failure. His renal function improved, and he was weaned from dialysis and discharged home on the 19th day.

**Conclusion:**

We should be aware of the possibility that severe renal function deterioration may occur in some patients with levetiracetam overdose. It is possible that clinicians underestimate the occurrence of this problem. In cases of acute renal failure in levetiracetam poisoning, induction of dialysis is beneficial.

## 1. Introduction

Levetiracetam (E Keppra) is a second-generation antiepileptic drug widely used for partial-onset seizures. Although there have been reports of adverse effects and overdose with levetiracetam, the symptoms were often minor, and the requirement for systemic management was rare.^[1]^ In this report, we describe a case of levetiracetam poisoning with renal failure requiring hemodialysis.

## 2. Case report

A 51-year-old man presented to our hospital with hypotension and disturbance of consciousness. The day before presentation, he had attempted suicide by taking an excessive amount of medication (namely levetiracetam, 200,000 mg; telmisartan, 480 mg; amlodipine, 30 mg; and brotizolam, 1.5 mg) and visited a doctor after confessing to his family that he had overdosed. Because his blood pressure was low, he was transferred to our hospital for systemic management.

The patient was an ethnic Japanese man who was born in Japan. He did not smoke tobacco, drink alcohol, or use illicit drugs. He had a history of hypertension, and cerebral arteriovenous malformations with associated symptomatic epilepsy. His medications comprised levetiracetam, telmisartan, amlodipine, and brotizolam. He had no known allergies and no distinctive family history.

On examination, the patient’s temperature was 37.2°C, blood pressure was 95/55 mm Hg, pulse was 86 beats per minute, respiratory rate was 32 breaths per minute, and oxygen saturation was 98% on room air. His consciousness score was E4V4M6, and cold extremities were noted. The remainder of the examination was normal. Laboratory testing revealed the following: blood urea nitrogen: 32.5 mg/dL (reference range, 8–20 mg/dL), creatinine: 3.17 mg/dL (reference range, 0.65–1.07 mg/dL), serum potassium: 3.2 mmol/L (reference range, 3.6–4.8 mmol/L), white blood cell count: 12,500/µL (reference range, 3300–8600/µL), and platelet count: 156,000/µL (reference range, 158,000–348,000/µL). Other laboratory test results were within their respective reference ranges. A radiograph of the chest and an electrocardiogram were normal. The patient was admitted to the intensive care unit for systemic management of levetiracetam poisoning.

**Figure 1.**
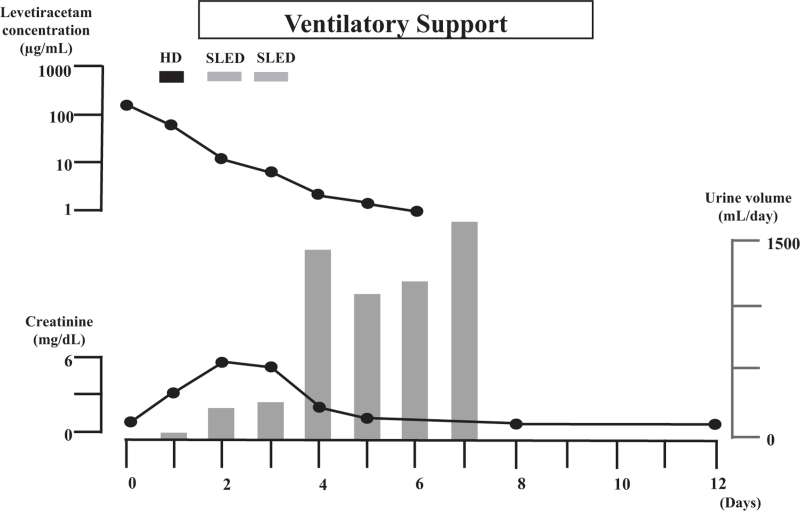
Patient’s clinical course. HD = hemodialysis; SLED = sustained low-efficiency dialysis.

The day after admission, he rapidly developed oliguria and elevated creatinine. We decided to introduce hemodialysis to treat the acute kidney injury and remove the levetiracetam. On the same hospital day, we started respiratory management with a ventilator under sedation because of difficulty during dialysis caused by the patient’s frequent body movements. Hemodialysis (blood flow rate, 150 mL/min; dialysate flow rate, 500 mL/min; dialysis time, 180 minutes) was performed on the second hospital day, and sustained low-efficiency dialysis^[2]^ (blood flow rate, 120 mL/min; dialysate flow rate. 300 mL/min) was performed on the 3rd and 4th hospital days for 360 and 240 minutes, respectively (Fig. 1).

Because the patient’s urine volume recovered, and his creatinine concentration decreased, we terminated dialysis on the 5th hospital day. No worsening of creatinine or decrease in urine volume occurred thereafter. On the 9th hospital day, the patient’s general condition had stabilized, and he was extubated. On the same day, lacosamide was introduced to prevent epileptic seizures. The patient was discharged home on the 19th hospital day.

The patient’s blood levetiracetam concentration was 188 μg/dL (reference range, 12–46 µg/mL; using liquid chromatography-mass spectrometry; BML, Inc., Tokyo, Japan) the day after discharge from the hospital, and the concentration decreased over time, as shown in Figure 1.

## 3. Discussion

Levetiracetam is a newer second-generation antiepileptic drug that inhibits the release of excitatory neurotransmitters by binding to synaptic vesicle protein 2A, potassium, and N-type calcium channels.^[3]^ Because levetiracetam is not metabolized in the liver, it can be used casually without concern regarding drug interactions, unlike conventional antiepileptic drugs. Additionally, levetiracetam can be used with adjustment of the patient’s renal function because it is excreted directly into the kidneys. Moreover, levetiracetam can be used in patients undergoing dialysis.^[3]^

In 2002, Barrueto et al^[4]^ reported the first case of poisoning by levetiracetam. The patient exhibited a decrease in the Glasgow coma scale score after excessive intake of oral levetiracetam, and management was performed with artificial respiration for protection and ventilatory support. The patient was extubated and transferred to the psychiatry department after awakening the next hospital day. Subsequent case reports described psychiatric,^[5]^ cardiovascular,^[6]^ and respiratory depression^[7]^ after excessive intake of levetiracetam, all of which resolved with symptomatic treatment, including discontinuation of levetiracetam and ventilatory support. Most cases resolved with no permanent disability.^[1]^

Recently, cases of acute kidney injury have been reported following introduction of levetiracetam at the conventional dose, suggesting that this drug may have adverse effects on renal function.^[8]^ Acute kidney injury is more likely to occur in patients with diabetes mellitus, hypertension, and infections.^[9]^ One report described histological evidence of interstitial nephritis.^[10]^ No specific treatment for this overdose exists; levetiracetam must simply be discontinued, and the patient must be treated according to his or her symptoms.

To the best of our knowledge, no reports have described similar cases of acute kidney injury following levetiracetam poisoning. Although hypotension could have affected the renal damage in this case, hypotension was present only in the early phase of the clinical course and was of relatively short duration. However, the renal impairment was sufficiently severe to require dialysis, and there was limited evidence that the effect of hypotension was sufficient to cause the kidney injury. Considering the recent reports of renal failure with standard doses of levetiracetam and with relatively low doses of other drugs, it is reasonable to consider levetiracetam as the main cause of the renal failure in this case. In this case, we assumed that levetiracetam could be effectively removed by hemodialysis based on its small distribution volume (0.7 L/kg) and low protein binding rate (<10%).^[11]^ Therefore, hemodialysis was performed, and the levetiracetam concentration decreased steadily. In addition, because there have been reports of cases of interstitial kidney disease associated with the introduction of levetiracetam,^[10]^ hemodialysis was continued for the treatment of acute kidney injury, and improvement of renal function was confirmed. In accordance with the Extracorporeal Treatments in Poisoning workgroup guidelines for dialysis methods,^[12]^ the hypotension was deemed manageable with the administration of cardiovascular agonists. Therefore, after the 1st round of intermittent dialysis, treatment for renal failure was continued with sustained low-efficiency dialysis.^[2]^

The mechanism of the renal injury, in this case, remains unclear. However, we cannot deny the possibility that interstitial nephritis occurred in our patient, similar to the findings in a previous case report of interstitial nephritis caused by the introduction of levetiracetam.^[10]^ Although there have been case reports of rhabdomyolysis and acute kidney injury associated with the introduction of levetiracetam,^[13]^ this is not considered applicable in our case because of the limited increase in the creatine kinase concentration.

If renal excretion of levetiracetam cannot be expected because of renal impairment, as in this case, hemodialysis should be considered in cases of levetiracetam poisoning as well as in cases of renal impairment at standard levetiracetam doses.

## 4. Conclusion

Levetiracetam is a relatively frequently used anticonvulsant. To our knowledge, there have been no reports of severe acute kidney injury requiring dialysis, as in this case; previous cases involved follow-up only. If acute kidney injury is confirmed after careful observation in cases of levetiracetam poisoning, dialysis could be effective.

## Acknowledgments

We thank Edanz (https://jp.edanz.com/ac) for editing a draft of this manuscript.
